# Evaluation of lab-defined syngas and acetate as substrates for H_2_ production with *Parageobacillus thermoglucosidasius* DSM 6285

**DOI:** 10.1007/s00253-025-13659-z

**Published:** 2025-12-09

**Authors:** Magda S. Ardila, Habibu Aliyu, Pieter de Maayer, Anke Neumann

**Affiliations:** 1 Section II: Electrobiotechnology, Institute of Process Engineering in Life Science, Karlsruhe Institute of Technology, 76131 Karlsruhe, Germany; 2 Section V: Biotechnology and Microbial Genetics, Institute for Biological Interfaces, Karlsruhe Institute of Technology, 76131 Karlsruhe, Germany; 3 School of Molecular & Cell Biology, Faculty of Science, University of the Witwatersrand, Johannesburg, 2000 South Africa

**Keywords:** *Parageobacillus thermoglucosidasius*, Hydrogen, Water-gas shift reaction, Syngas, Acetate

## Abstract

**Abstract:**

*Parageobacillus thermoglucosidasius* is a carboxydotrophic microorganism that produces H_2_ through the water-gas shift (WGS) reaction, using carbon monoxide (CO) as the main substrate. CO is a common constituent of syngas, alongside CO_2_, H_2_, O_2_, and other gases. The facultatively anaerobic nature of *P. thermoglucosidasius* is particularly pertinent for hydrogenogenesis from O_2_-containing syngas. Here, we evaluated the effects of different syngas compositions (5, 12, and 20% of H_2_ gas, with constant CO and CO_2_; 10, 30, and 50% CO gas with constant CO_2_ and H_2_) on hydrogenogenesis at the bioreactor scale. Electron balance analysis showed that 88–91% of electrons coming from CO were converted into H_2_, regardless of the gas composition. The presence of H_2_ in different compositions had no inhibitory effect on hydrogen production rate (HPR), and the maximum HPR corresponded to 13.65 L H_2_ L⁻^1^ day⁻^1^ in fermentations containing 30% CO. A carbon source, other than CO, is needed for biomass formation of *P. thermoglucosidasius*. Acetate was shown to be the primary intermediate metabolite of glucose metabolism, but could also be used as an initial carbon source for biomass generation. When this carbon source was used, most electrons from CO were converted to H_2_, demonstrating that this organic acid can be used as an effective alternative to glucose for H_2_ production with *P. thermoglucosidasius*.

**Key points:**

• *Evaluation of lab-defined syngas at different compositions for H2 production with P. thermoglucosidasius at the bioreactor scale*.

• *Hydrogen presence in the headspace was not inhibiting for subsequent H2 production*.

• *Acetate can replace glucose to generate biomass when growing P. thermoglucosidasius*.

**Supplementary Information:**

The online version contains supplementary material available at 10.1007/s00253-025-13659-z.

## Introduction

Dwindling fossil fuel reserves and the increase in atmospheric CO_2_ emissions as a result of their combustion are driving the development of alternative energy sources to meet growing global energy demand (Achakulwisut et al. 2023). Renewable production approaches, such as electrolytic, thermolytic, and photo-electrolytic water-splitting technologies, as well as biological (biomass) transformations, may provide a sustainable means for the production of H_2_, an attractive fossil fuel alternative (Nikolaidis and Poullikkas 2017; Balachandar et al. 2020; Martins et al. 2021). The latter involves the use of bacteria and algae to produce H_2_ photolytically (from water and sunlight), or through dark fermentation, using biomass or organic acids through hydrogenase or nitrogenase enzyme systems (Nikolaidis and Poullikkas 2017; Balachandar et al. 2020). In addition to the existing biological processes for H_2_ production, carboxydotrophic microorganisms that produce H_2_ and CO_2_ through the water-gas shift (WGS) reaction, using carbon monoxide (CO) and water as substrates, are a potential option for CO-based H_2_ production (Mohr et al. 2018; Martins et al. 2021). The thermophilic facultatively anaerobic bacterium *Parageobacillus thermoglucosidasius* is a metabolically versatile, biotechnologically relevant microorganism that can perform the WGS reaction in the presence of low levels of oxygen (Mohr et al. 2018, 2019; Mol et al. 2024). Syngas, gas mixtures comprised primarily of CO, H_2_, and CO_2_, are produced through the industrial gasification of coal, biomass, or natural gas and may serve as a good source for WGS-driven hydrogenogenesis, but often contain traces of oxygen, making *P. thermoglucosidasius* an attractive candidate for syngas-derived hydrogen production (Mol et al. 2024). In addition to syngas, steel mill off-gases could also be potentially used for H_2_ production (Collis et al. 2021). Previous studies have shown that this bacterium was able to perform the WGS reaction with complex syngas mixtures and that this gas substrate led to a shorter lag phase before the commencement of hydrogenesis (Mol et al. 2024), compared to when more purified (CO and N_2_) gas mixtures were used (Aliyu et al. 2021).

In previous work, we evaluated the effect of increasing CO, N_2_, and H_2_ partial pressures at the bottle scale, concluding that increasing the CO partial pressure to 3.0 bar inhibited H_2_ production, while rising N_2_ and H_2_ partial pressures had a positive effect (Ardila et al. 2024). Pressure was evaluated as a process parameter, as the experiments allowed for assessing the total pressure of the system and comparing the different partial pressures of the gas mixtures used (Ardila et al. 2024). In comparison, the present work aimed to evaluate the effects of increased CO and H_2_ percentages in a gas mixture containing CO, CO_2,_ and H_2_, at ambient pressure, on hydrogenogenesis at the bioreactor scale with continuous gas flow and pH regulation. Scaling up to bioreactor systems is essential for gaining insights into fermentation processes, as bottle fermentations offer limited control over critical parameters such as pH, temperature, and gas flow rates. Furthermore, gas-liquid mass transfer can be enhanced through mixing and sparging systems in bioreactors, whereas in bottles, gas diffusion is achieved solely by stirring (Chezeau et al. 2019; Liu et al. 2019).

The CO content of the syngas mixtures evaluated in serum bottles was 17% and 38% (Mol et al. 2024). However, syngas composition can vary widely depending on factors such as production method, reactor design, gasifying agent, and feedstock type (Benevenuti et al. 2021). Typical compositions range from 5 to 40% H_2_, 7 to 40% CO, 2 to 70% N_2_, 10 to 40% CO_2_, and 0.2 to 12% CH_4_ (Benevenuti et al. 2021). In addition to the presence of impurities, the H_2_/CO ratio is a critical parameter that must be considered before integrating syngas into other processes, especially given recent efforts to produce CO-rich syngas for specific applications (Chan et al. 2021; Benevenuti et al. 2021). Evaluating gas mixtures with higher CO and H_2_ contents is essential to understand the limits of the WGS reaction with *P. thermoglucosidasius* and to explore the potential for detoxifying CO-rich mixtures.

In addition to using syngas as a substitute for pure CO in bioreactor-scale fermentations, glucose in the medium must also be replaced to enable a sustainable scale-up process. Acetate, or more specifically its protonated form acetic acid, can be found in biomass gasification waste streams (Harahap and Ahring 2023). Microorganisms such as acetogenic bacteria that metabolize syngas often produce acetate as an intermediate or end product, such as acetogenic bacteria, which can convert CO and CO_2_ into acetate via the Wood-Ljungdahl pathway (Redl et al. 2017; Arantes et al. 2020). This organic acid is also produced by *P. thermoglucosidasius* as a metabolite during fermentation, following glucose depletion, and is later consumed during the fermentation (Aliyu et al. 2021). Therefore, a set of fermentations was designed to evaluate acetate as a carbon source and its effects on H_2_ production in *P. thermoglucosidasius*.

## Materials and methods

### Microorganisms and media

*P. thermoglucosidasius* DSM 6285 was acquired from the Deutsche Sammlung von Mikroorganismen und Zellkulturen (DSMZ, Braunschweig, Germany) and was conserved in glycerol (80%) stocks at −80 °C. Routine cultivation of *P. thermoglucosidasius* DSM 6285 was performed in modified Luria Bertani (mLB) medium (Mohr et al. 2019). Bioreactor fermentations were undertaken in modified ammonium sulfate medium (mASM) containing 1 g/L glucose (Greening et al. 2016; Ardila et al. 2025). For the acetate fermentations, glucose (1 g/L) was replaced with acetate at the same concentration (16.7 M = 1 g/L).

### Inoculum preparation

A volume of 300 µL of glycerol stock was added to 200 mL of mLB medium in 500-mL shake flasks and grown under aerobic conditions at 60 °C, and rotation at 120 rpm in an Infors Thermotron (Infors Thermotron, Infors AG, Bottmingen, Switzerland). After 14 h, a calculated volume of the inoculum was added to the reactors to achieve an initial absorbance (OD_600_) of 0.1 for a total volume of 1 L.

### Experimental setup

Each fermentation was performed in two bioreactors of 2.5 L capacity (Minifors, Infors AG, Bottmingen, Schweiz) with a 1 L working volume. The growth conditions were maintained as reported previously (Ardila et al. 2025), with stirrer speed set to 500 rpm, temperature to 55 °C, and pH to 6.8; pH was controlled using a pH probe (Easyferm plus, Hamilton, Switzerland) and with the help of a peristaltic pump connected to the reactor system providing NaOH (1 M) and H_2_SO_4_ (1 M). For the syngas fermentation, an anaerobization step was performed to ensure no oxygen was present in the reactors before inoculation. This was achieved by flushing the reactors overnight with nitrogen (N_2_) gas. Two hours before the addition of the inoculum, the gas mixture was set through mass flow controllers, with a flow rate of 200 mL min^−1^ and the gas compositions outlined in Table 1. The two-phase fermentation using acetate as an additional substrate was performed as described before (Ardila et al. 2025). The aerobic phase (Acetate P1) had a continuous flow rate of 100 mL min^−1^ of air and CO for 24 h, while the anaerobic phase (Acetate P2) had an 80 mL min^−1^ flow of a mixture of 80% nitrogen and 20% CO (Table 1).

**Table 1:** Gas mixtures for each fermentation, with increasing CO (1–3) and H_2_ (4–6) percentages

Fermentation	H_2_ (%)	CO (%)	CO_2_ (%)	N_2_ (%)
CO-1	15	10	15	55
CO-2	15	30	15	35
CO-3	15	50	15	20
H_2_−4	5	20	15	60
H_2_−5	12	20	15	53
H_2_−6	20	20	15	45
Acetate P1*	-	10	-	-
Acetate P2	-	20	-	80

*In Acetate P1, there was also 90% air

### Analytical methods

Online measurement of the gas content in the headspace of the reactor was performed using a 3000 Micro GC gas analyzer (Inficon, Switzerland) connected with Molsieve and PLOT Q columns for data acquisition. Liquid samples were withdrawn daily, and absorbance (OD_600_) readings were confirmed using an Ultrospec 1100 pro spectrophotometer (Amersham Biosciences, Uppsala, Sweden). HPLC analyses were performed on the liquid samples using the Agilent 1100 series HPLC system (Agilent Technologies, Waldbronn, Germany), equipped with a 50-mm pre-column (model Rezex ROA-Organic Acid H+ 8% Guard Column) and a 300-mm separation column (model Rezex ROA-Organic Acid H+ 8%), together with a wavelength detector and refractive index detector. Operational parameters were 55 °C for column temperature, flow rate of 0.6 mL min ^−1^, injection volume of 10 µL, a mobile phase of 5 mM H_2_SO_4_, and a duration of 40 min per sample. ChemStation software (Agilent Technologies) was used for data acquisition and analysis. Gas composition was calculated according to the ideal gas law, as described before (Mohr et al. 2018). The hydrogen production rate (HPR) was calculated based on the difference between H_2_ in the outflow and the H_2_ in the inflow; more information can be found in the supplementary material. The electron selectivity was used to show the electron flux in the process. This was calculated from the electron mole (e^−^ mol) of each compound, calculated from the quantities of each compound (mmol), and a conversion factor to electrons based on the oxidation state of each element. The calculation of the electron balance takes into account the electrons from CO, glucose, and acetate (once acetate starts to be consumed). Additional information on the calculations can be found in Table S1.

## Results

### Effects of different CO concentrations on H_2_ production

**Fig. 1:**
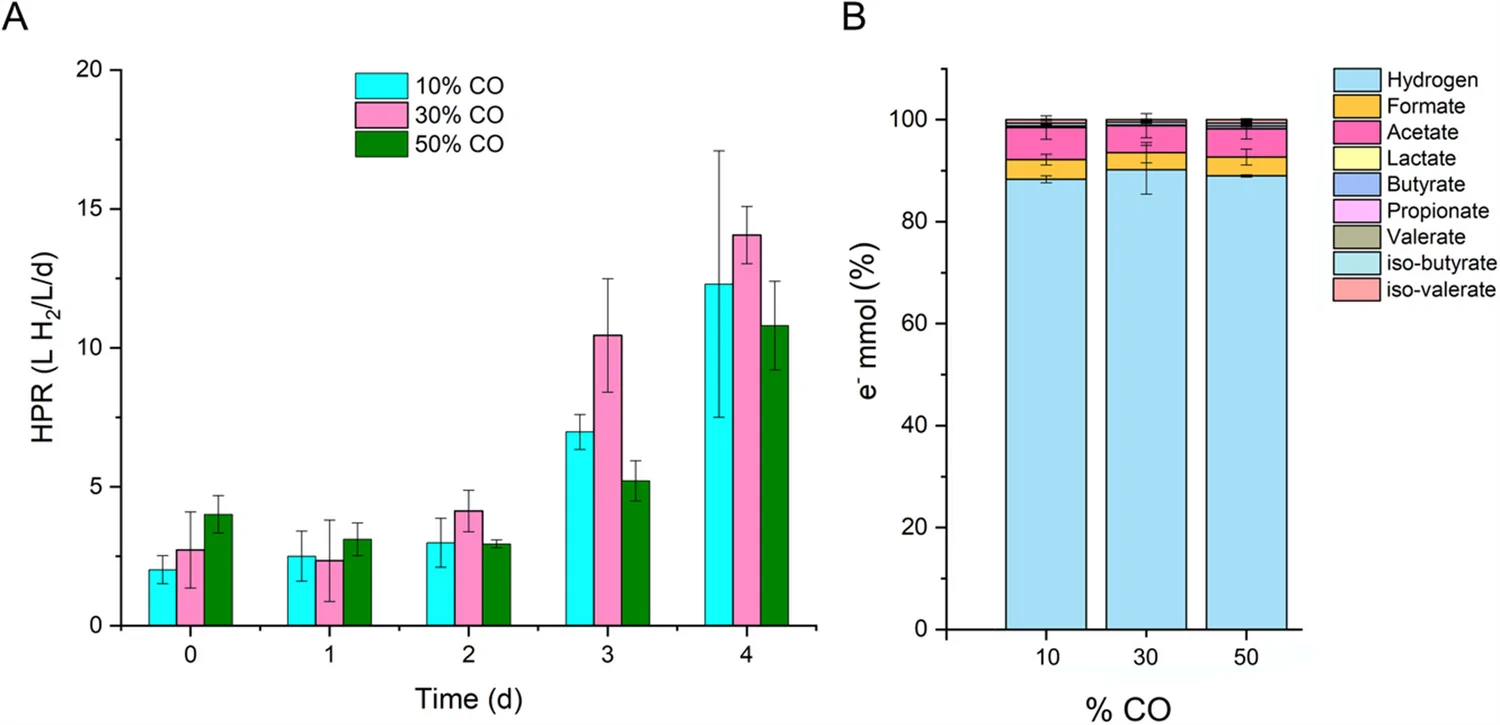
**A** HPR with increasing percentage of CO. **B** Selectivity of the process in terms of electron balance at 4 days. The represented data is the average of two reactors, and the error bars indicate the minimum and maximum values

The increase of CO was performed to evaluate possible inhibition on the hydrogen production rate by a high CO percentage within the gas mixture. The HPR is given in liters of H_2_ produced per liter of growth media per day. Evaluation of the HPRs at the three different CO percentages showed that H_2_ produced was similar until day 2 and increased to 14.1 L H_2_/L/day on day 4 (Fig. 1A). Unconverted CO was detected in the gas outflow (Fig. S1).

Analysis of the electron balance within the CO percentages evaluated was similar, as 88–90% of electrons coming from CO, glucose, and acetate were converted into H_2_, with acetate as the second main product, followed by formate (Fig. 1B). Other metabolites such as lactate, butyrate, propionate, and valerate were produced in lower proportions (< 1%).

**Fig. 2:**
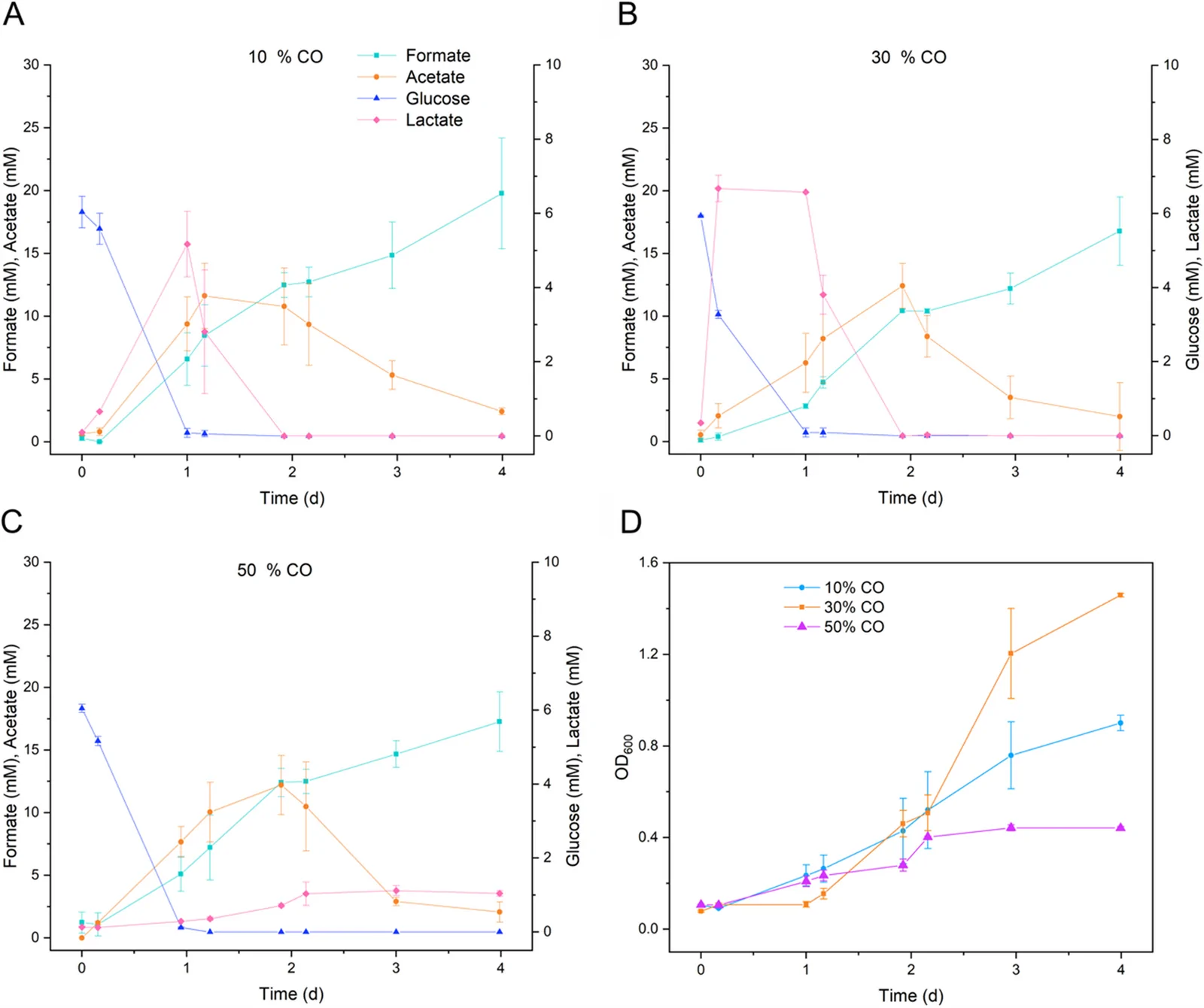
**A** Metabolite concentration during the fermentations at increasing CO percentages. **B** Growth of *P. thermoglucosidasius* in terms of absorbance (OD_600_). The data shown represent the average of two reactors, with error bars indicating the minimum and maximum values

Glucose (5.5 mM) present in the media was fully consumed by the first day of fermentation at all three CO levels, while formate was cumulatively produced in all CO percentages evaluated (Fig. 2A). Acetate production was similar reaching 11.6, 12.4, and 12.2 mM at 10, 30, and 50% CO, respectively, by day 2, before being almost completely consumed by day 4. In comparison, lactate accumulation showed a similar trend at CO levels of 10%, 30%, and 50%, reaching concentrations of 5.2, 6.6, and 1.1 mM, respectively. At 10% and 30% CO, lactate was then fully consumed within two days.

The greatest biomass (OD_600_) was attained at 30% CO. After a long lag phase of nearly 1 day, the absorbance started to increase, reaching a maximum of 1.5 at day 4 post-inoculation (Fig. 2B). At 10% CO, absorbance started to increase from 0.16 days post-inoculation and reached an OD_600_ of 0.9 by the end of the fermentation (4 days). After 1 day, the absorbance at 50% CO increased to 0.1 and reached 0.4 at day 2 of fermentation, remaining constant until day 4.

### Effects of increased H_2_ percentages in the gas mixture on H_2_ production

**Fig. 3:**
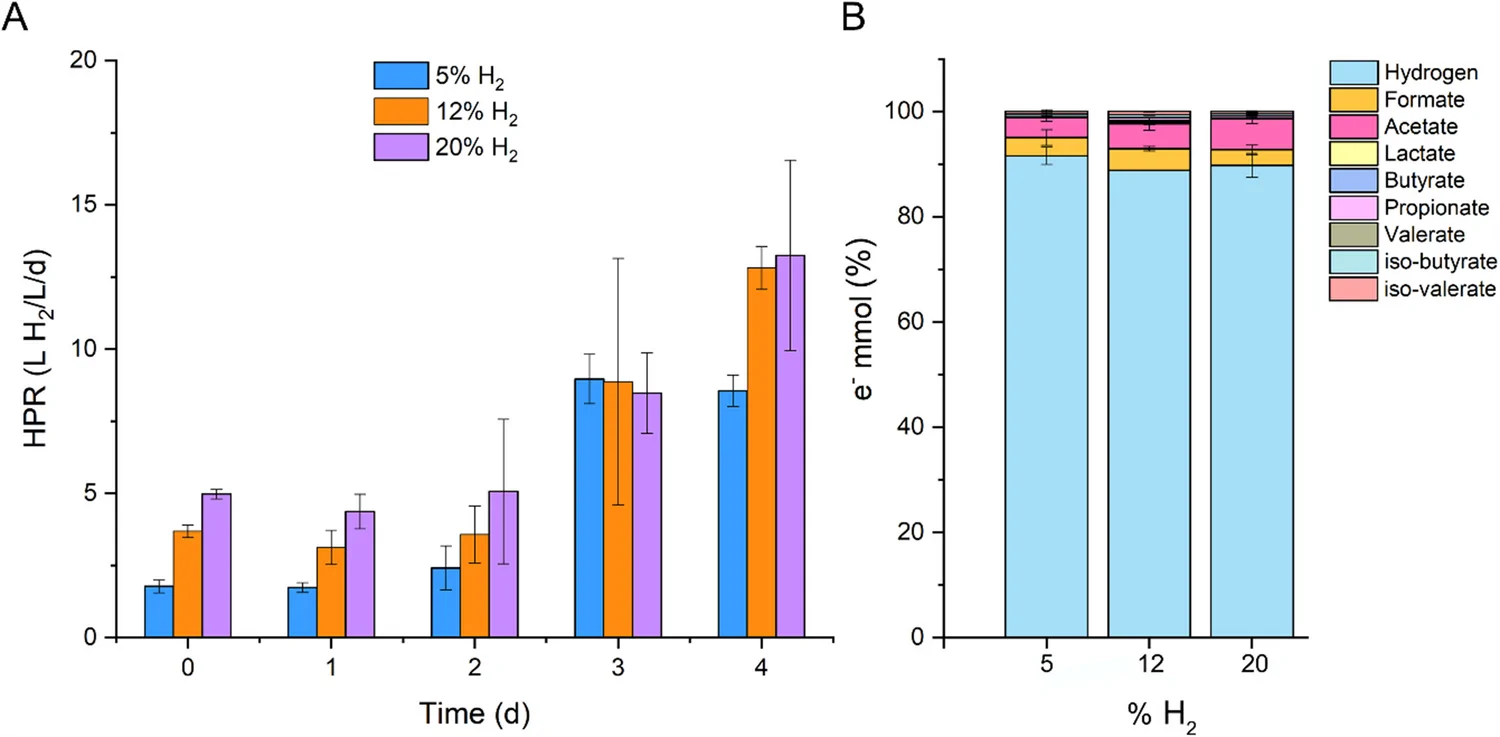
**A** HPR with increasing H_2_ percentage. **B** Selectivity of the process in terms of electron balance at 4 days. The represented data is the average between two reactors and the error bars indicate the minimum and maximum values

Considering H_2_ being the product of the WGS reaction, increased H_2_ would be expected to inhibit the reaction. While there were no noticeable differences in hydrogen production 2 days post-inoculation (Fig. S2), at 3 days post-inoculation, the HPRs achieved with 5%, 12%, and 20% were similar (Fig. 3A). By day 4, the HPR was maintained with the 5% H_2_, and similar HPR values were observed, in the range of 12–13 L H_2_/L/day with 12 and 20% H_2_.

As observed for the experiments with increasing CO, most of the electrons from CO went to H_2_ production (Fig. 3B). When H_2_ was increased from 5 to 20%, acetate tended to increase.

**Fig. 4:**
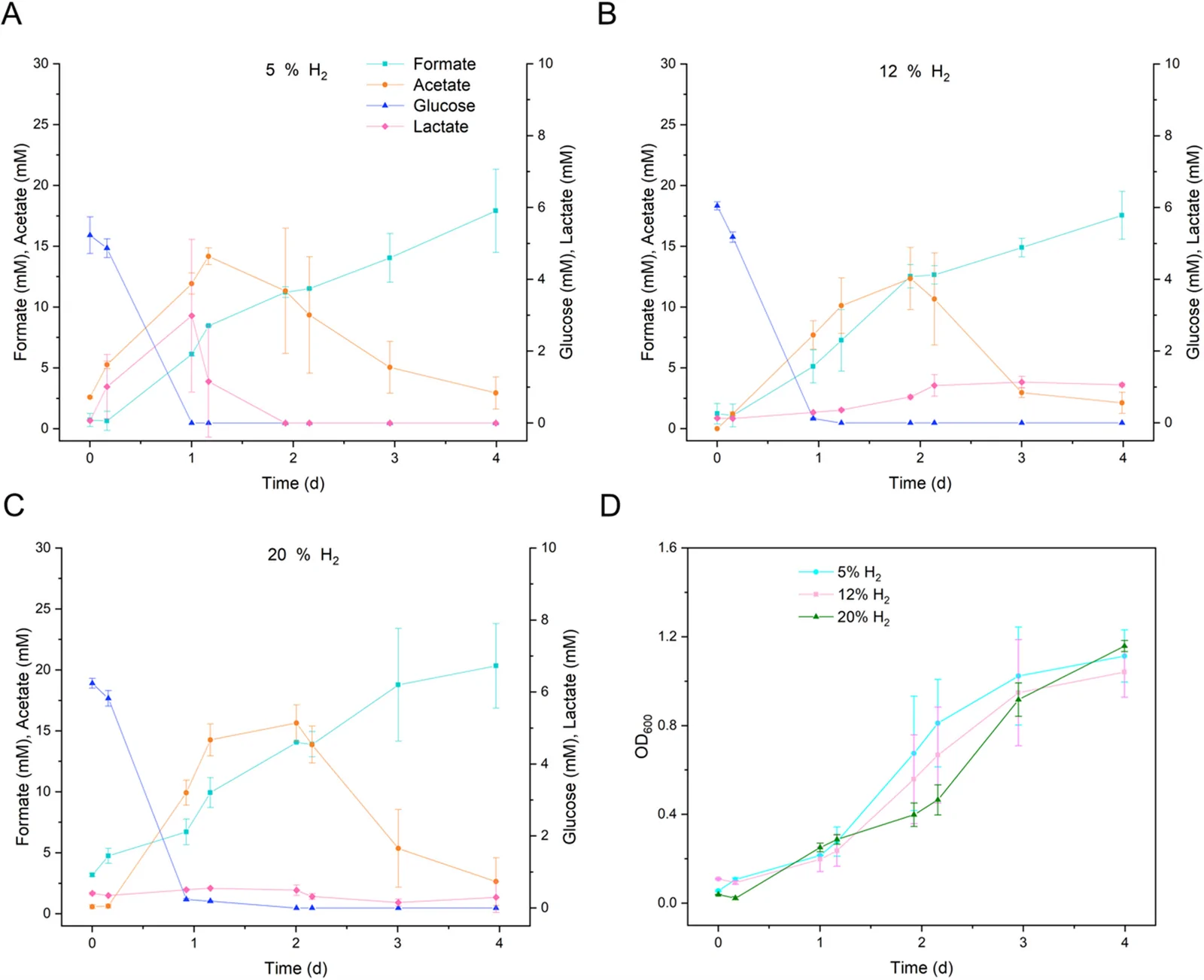
**A** Metabolite concentration during the fermentations at different H_2_ percentages. **B** Growth of *P. thermoglucosidasius* in terms of absorbance (OD_600_). The data shown represent the average of two reactors, with error bars indicating the minimum and maximum values

Similar to the trend observed with increasing CO, glucose was consumed (5.5 mM) on the first day when H_2_ was increased. Formate had a tendency to increase in all H_2_ levels, up to 20 mM with 20% H_2_. Acetate production increased up to 17 mM at 12% H_2_ within 1 day before being consumed by day 4; a similar trend was observed with 5% H_2_ (Fig. 4A). Maximum lactate production reached 3 mM at 5% H_2_.

Similar biomass accumulation was observed at 5 and 12% H_2_. Even though with 20% H_2,_ an OD_600_ value 50% lower than that with 5% H_2_ was observed 2 days post-inoculation, all the H_2_ levels evaluated resulted in similar OD_600_ values (~ 1.2) by 4 days post-inoculation (Fig. 4B).

### Acetate as substrate for *P. thermoglucosidasius*

**Fig. 5:**
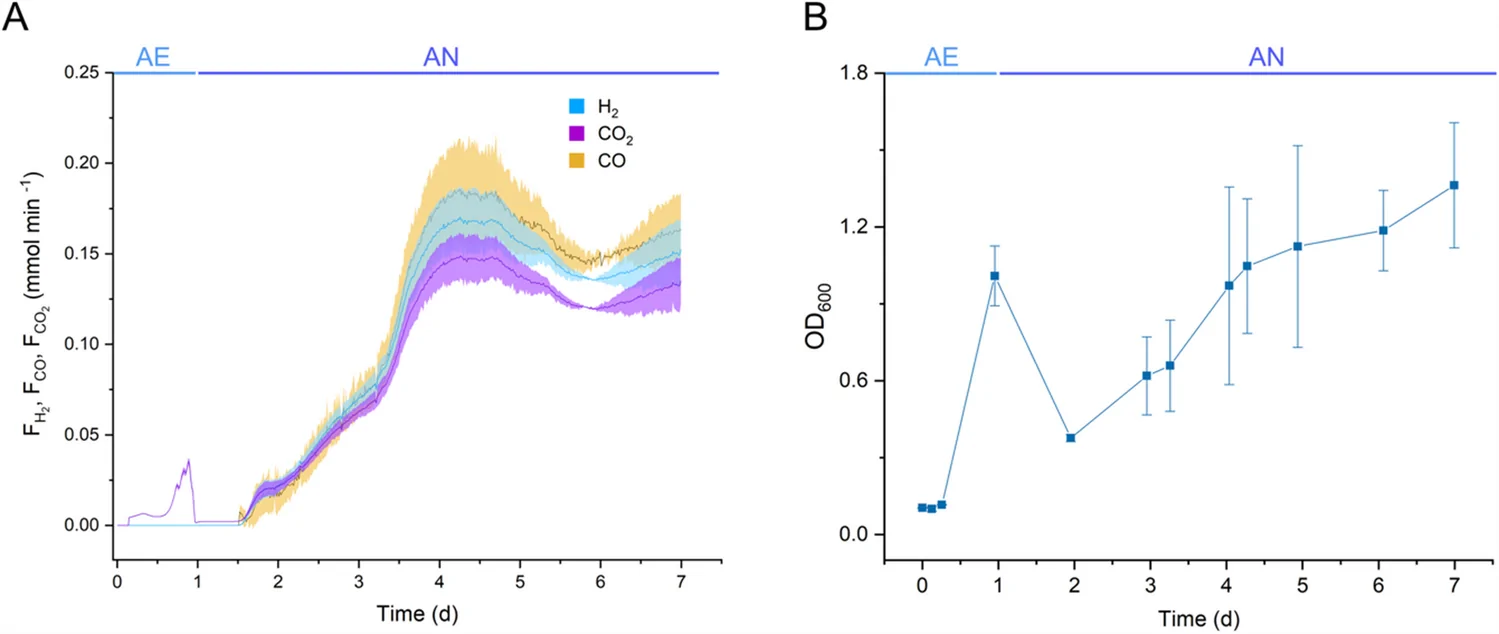
Batch fermentation with acetate as substrate. **A** H_2_ and CO_2_ production rate (mmol min^−1^) and CO consumption rate (mmol min^−1^) with the standard deviation indicated by the colored regions. **B** Growth indicated by absorbance OD_600_. The aerobic (AE) phase is denoted in light blue, while the anaerobic (AN) phase appears in dark blue. The gas exchange occurs after 1 day of fermentation. The data shown represent the average of two reactors, with error bars indicating the minimum and maximum values

In batch fermentations using acetate as an additional carbon source, CO uptake followed a slightly delayed pattern (Fig. 5A). The two-phase fermentation starts aerobically with a mixture of air and CO, followed by an anaerobic phase with CO and N_2_. This was done to increase the biomass before the gas exchange. Additionally, *P. thermoglucosidasius* biomass decreased from an absorbance (OD_600_) of 1.0 ± 0.1 to 0.37 ± 0.01 after gas exchange. However, the OD recovered in correlation with H_2_ production, which began 1.5 days post-inoculation and continued until the end of fermentation (Fig. 5B). The decrease in OD observed after the gas exchange has been observed before, as the microorganism needs to adapt to the anaerobic conditions and start CO consumption (Ardila et al. 2025).

Acetate consumption began around day 1 of the fermentation, but CO consumption was initiated at approximately 1.5 days. H_2_ production increased gradually in this case, reaching its peak HPR of 0.167 ± 0.017 mmol min⁻^1^ at day 4. In contrast, in a previous study, when glucose was the additional carbon source, *P. thermoglucosidasius* began consuming CO after 1 day, following gas exchange (Ardila et al. 2025). H_2_ production via the WGS reaction started concurrently, reaching a maximum HPR of 0.144 ± 0.002 mmol min⁻^1^ by day 4. This indicates that although the onset of WGS activity was slower with acetate, it led to a slightly higher maximum HPR.

**Fig. 6:**
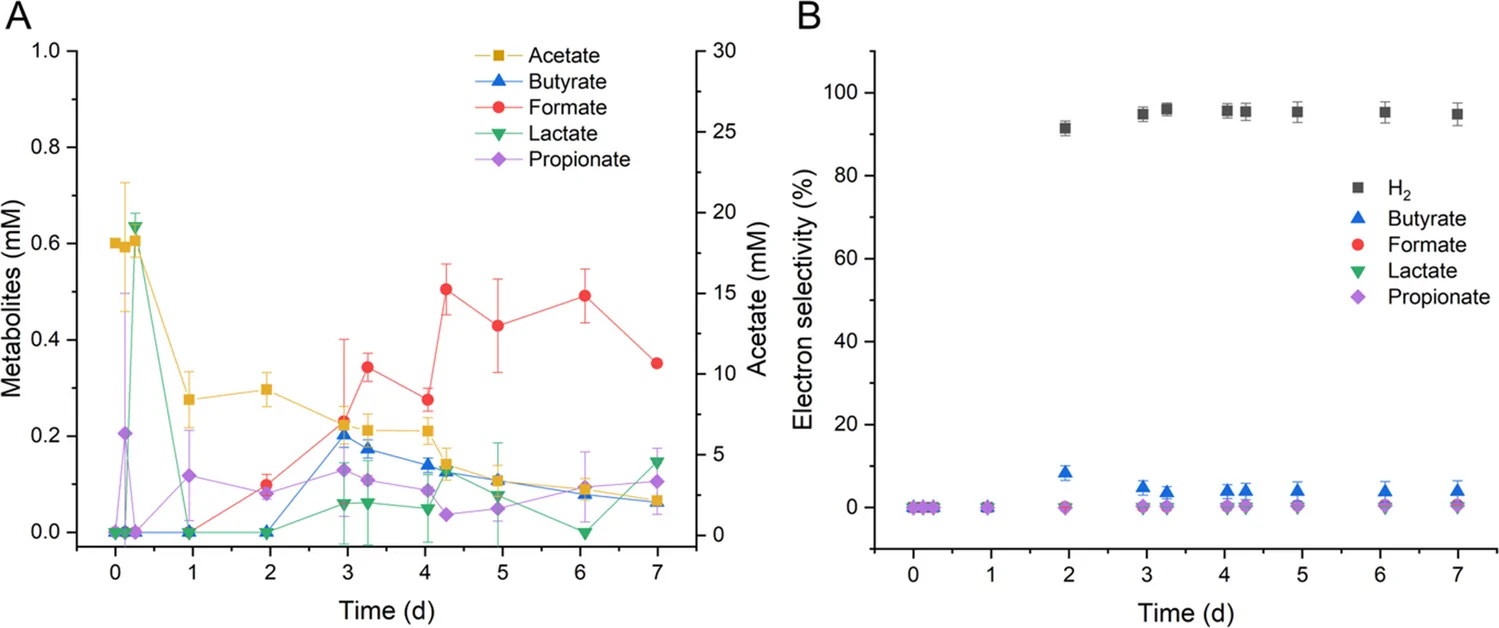
**A** Metabolites concentration during the fermentation with *P. thermoglucosidasius* DSM 6285*.* **B** Electron selectivity along the fermentation. The data shown represent the average of two reactors, with error bars indicating the min and max values

The metabolite production profiles differed significantly between glucose and acetate fermentations. The acetate fermentation resulted in markedly reduced production of most organic acids (Fig. 6A). Notably, butyrate became the predominant product once acetate consumption began, reaching a production rate of 0.2 mmol day⁻^1^ within just 0.16 day. This suggests a more efficient metabolic response, with carbon flux favoring butyrate and hydrogen production over a wider spread of by-products. On the other hand, in the glucose batch fermentations, from a previous study, *P. thermoglucosidasius* produced a broad mix of organic acids, particularly formate, lactate, acetate, and propionate (Ardila et al. 2025). Formate was the dominant product, and acetate production was closely associated with glucose metabolism, decreasing once the substrate was depleted.

Electron selectivity analysis revealed important differences in how electrons from CO and the carbon substrate were distributed. During acetate-fed fermentations, 91.4% electron selectivity toward H_2_ was reached by 2 days and maintained thereafter (Fig. 6B). However, acetate fermentation also channeled a small but notable portion of electrons, around 8.3%, into butyrate production at 2 days. This tighter distribution of electron flow toward fewer end-products implies a more efficient conversion of substrates into targeted outputs, particularly hydrogen and butyrate. A similar trend was observed in a previous study, where glucose fermentation was performed in similar conditions; electrons were initially routed toward acetate, then shifted toward H_2_ production following gas exchange (Ardila et al. 2025). By day 2, 79% of electrons were directed toward H_2_ production, and this value remained stable at around 90% by day 3.

Carbon recovery during acetate fermentation—calculated based on the carbon from the substrates (CO and acetate) incorporated into product formation—exceeded 80%, indicating that the majority of the carbon was successfully converted into products such as formate, lactate, butyrate, propionate, valerate, iso-butyrate, and iso-valerate (Fig. S3). The ratio of CO_2_ produced to CO consumed ranged from 0.8 to 1.0, suggesting that most of the carbon derived from CO was converted into CO_2_ (Fig. S4). Additionally, the data indicate that acetate was primarily converted into biomass and other organic acids, including formate, lactate, butyrate, propionate, valerate, iso-butyrate, and iso-valerate.

## Discussion

Understanding the impact of gas composition on microbial growth and metabolism is crucial for optimizing fermentation processes. While the total gas flow in the experiments remained constant, the CO and H_2_ percentages were varied to assess potential substrate or product inhibition. Additionally, scaling up to a bioreactor is necessary, as bottle fermentations lack precise control over different growth parameters. Scaling up from bottle fermentations to batch and semi-continuous systems provided additional insight into the limitations of the process (Ardila et al. 2025). An increase in CO positively influenced growth up to 30% CO. However, further increasing CO to 50% led to a 42% reduction in growth by 3 days. The difference in growth has been described with a non-hydrogenogenic strain, *Parageobacillus toebii* DSM 14590^T^, when compared to other strains producing H_2_ (Mohr et al. 2018). This suggests that there might be additional growth derived from the production of hydrogen.

A recent study investigating *Rhodospirillum rubrum* in a bioreactor found that pCO became inhibitory at 1.0 atm, where the growth rate decreased from 0.058 to 0.040 h⁻^1^ as pCO increased from 0.2 to 1.0 atm. Despite this, H_2_ production improved significantly, rising from 1.81 to 4.88 mol under the same conditions (Rodríguez et al. 2021).

Overall, the increase in CO percentages did not affect HPRs. This observation aligns with our previous study, which examined syngas fermentation in bottle experiments using different gas compositions (Mol et al. 2024). In that study, CO depletion occurred earlier with a gas mixture containing 17.28% CO, whereas a higher CO concentration (38.01%) left residual CO in the headspace. Although this did not completely inhibit H_2_ production, it delayed the onset of hydrogenogenesis (Mol et al. 2024). As mentioned above, the composition of syngas depends on factors such as production methods and feedstocks, which influence the H_2_/CO ratio and result in a wide variety of CO-containing mixtures (Benevenuti et al. 2021). In this study, we showed that gas mixtures with up to 50% CO can be effectively detoxified and enriched in H_2_ using continuous CO-fed fermentation.

One of the parameters to improve H_2_ production at the bioreactor scale would be to evaluate different CO flow rates or adapt *P. thermoglucosidasius* to higher CO percentage in syngas. In an adaptive laboratory evolution study with *R. rubrum*, the evolved strains showed up to 50% additional H_2_ production and a reduced lag phase, compared to the parental strain, demonstrating their adaptation to gas photofermentation (Hernández-Herreros et al. 2024). Contrariwise, this could also cause a decrease in the conversion efficiency, as described for *Carboxydothermus hydrogenoformans* (Haddad et al. 2014). According to Henry’s law, the amount of dissolved gas is proportional to its partial pressure in the gas phase (Sander 2023). The low Henry’s solubility constant of CO (9.7 × 10^–6^ mol/m^3^ Pa) indicates its poor solubility in aqueous systems, thereby restricting the concentration of dissolved CO accessible to microbial cells (Do et al. 2007; Sander 2023). Consequently, increasing the CO partial pressure or the total system pressure is required to enhance CO availability (Ardila et al. 2024). This has to be considered when conditioning syngas with higher CO levels.

Increasing H_2_ to 20% in a gas mixture containing CO and CO_2_ was not detrimental to H_2_ production, with a maximum HPR of 13.2 L H_2_ L⁻^1^ day⁻^1^ at 3 days at this H_2_ percentage. In another study, increasing pH_2_ up to 1.52 bar increased ethanol production and hydrogen uptake rate in a syngas fermentation performed with *Clostridium ljungdahlii* (Perret et al. 2024). However, a further increase of H_2_ had negative effects, possibly due to inhibition of an enzymatic reaction above a critical equilibrium concentration of H_2_ in the liquid phase (Perret et al. 2024). In this study, an increase in H_2_ percentage led to comparable HPR values, considering the variability among reactors. This could suggest that the media was saturated with H_2_, the maximum theoretical solubility of H_2_ in water at 50 °C and 1 atm reported is 0.0127 cm^3^/g (0.52 mM), which is affected by the concentration of salts in the media and the partial pressure of the gas (Crozier and Yamamoto’ 1974; Baranenko and Kirov 1989).

Previous anaerobic fermentations with *P. thermoglucosidasius* DSM 6285 showed that the onset of the WGS reaction was faster and had shorter lag phases for H_2_ production when syngas was used compared to pure mixtures of CO and N_2_ (Mol et al. 2024). This was also encountered in our fermentations using syngas compared to previous work with a two-phase system to change from aerobic to anaerobic conditions, where CO consumption started after 0.5 days from the gas exchange on a batch fermentation, reaching a maximum consumption rate at 4 days (Ardila et al. 2025). A possible reason could be the additional time cells need to adapt to the shift from the aerobic to the anaerobic phase. In contrast, syngas fermentation may allow for a more seamless metabolic transition due to its composition, reducing the lag phase. Additionally, the presence of H_2_ in the syngas mixtures can lead to enzyme activation and metabolic adaptation in the strain (Esquivel-Elizondo et al. 2017).

As demonstrated by the fermentations with the different CO and H_2_ levels (this study) and previous experiments (Aliyu et al. 2021), acetate is the primary metabolite when glucose is used as a carbon source for hydrogenogenic fermentation. Here, we evaluated the use of acetate to serve as a carbon source (alongside CO). Previous evaluation with glucose achieved a specific H_2_ production rate of 2164 mmol H_2_ g CDW^−1^ (Ardila et al. 2025). Using 16.6 mM acetate, a specific H_2_ production rate of 2303 mmol H_2_ g CDW^−1^ was achieved, translating into a 6% increase in the specific H_2_ production rate with the latter substrate. Under anaerobic conditions, butyrate production from acetate could occur through microbial chain elongation, as reported for *Clostridium kluyveri*; this process would, however, require a reducing co-substrate such as ethanol, lactate, or H_2_ (Joshi et al. 2021). Formate production from acetate requires low hydrogen partial pressures and typically occurs through syntrophic interactions with hydrogenotrophic methanogens (Hattori 2008). Therefore, it is more likely that for *P. thermoglucosidasius*, anaerobic reduction or oxidation of formate is linked to the presence of formate dehydrogenase genes (Mohr et al. 2018).

High acetate concentrations can inhibit microbial growth due to the uncoupling effect of organic acids, i.e., acetic acid can diffuse across the cell membrane and affect the osmotic pressure (Pinhal et al. 2019). In a previous study, the maximum acetate production reported was 12.1 mM (Mol et al. 2024). There is no known inhibitory concentration for *P. thermoglucosidasius* until now. A concentration of 18.10 mM was used in the current study. Additionally, the inhibitory concentration also depends on the pH value. It has been reported that lowering pH to 5.5 can increase the undissociated acetic acid, which can be inhibiting for methanogens (Robazza et al. 2024). In the present fermentation with *P. thermoglucosidasius*, acetate was predominant because the pH was kept at 6.8, above the pKa of acetic acid (4.75), thereby avoiding inhibition of the microorganism growth by the undissociated form (Trček et al. 2015). Therefore, process optimization, evaluating different substrate concentrations, gas flow rates, and agitation speeds, is required when using acetate as a carbon source in a semi-continuous fermentation or a chemostat (Younesi et al. 2008). Gradual feeding of acetate or using acetate-tolerant strains can be a solution for further upscaling strategies (Najafpour et al. 2004).

## Conclusions

Higher percentages of CO led to a delayed onset of hydrogenogenesis. H_2_ presence in different compositions up to 20% H_2_ had no inhibitory effect on HPR; therefore, H_2_ does not inhibit the water-gas shift reaction. Electron flow was primarily directed toward hydrogen production, with the remainder contributing to the formation of organic acids. The use of a clean, syngas-like gas mixture, free from common microbial inhibitors such as tars, ammonia, hydrogen sulfide, particulates, among others (Ramachandriya et al. 2016), provided a controlled baseline for evaluating microbial performance and scaling up the process. Additionally, acetate proved to be an effective alternative to glucose for biomass production during the aerobic phase, offering several advantages due to its direct entry into central metabolism and its lower cost. These characteristics make acetate a promising substrate for hydrogenogenic fermentation.

## Supplementary Information

Below is the link to the electronic supplementary material.

ESM 1 (PDF 792 KB)

## Data Availability

The dataset is included within the work.
