# Supplementary material for: Evaluation of lab-defined syngas and acetate as substrates for H2 production with Parageobacillus thermoglucosidasius DSM 6285
Source: Appl Microbiol Biotechnol. 2025 Dec 9;109(1):261. doi: 10.1007/s00253-025-13659-z (PMC12696036; doi:10.1007/s00253-025-13659-z)
Supplement: Supplementary file 1 — (PDF 792 KB) [file 253_2025_13659_MOESM1_ESM.pdf]

Additional file 1

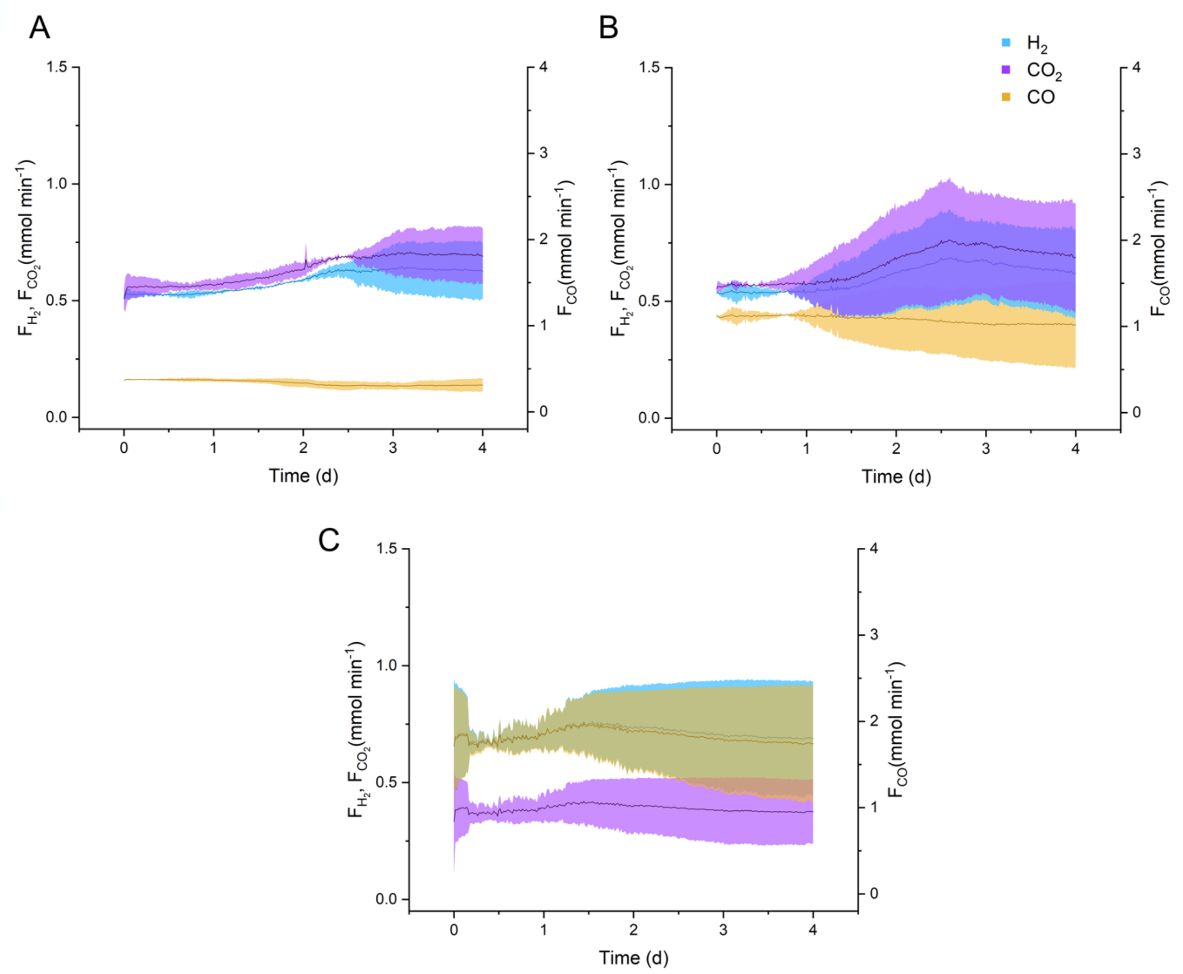

Figure S1. Syngas fermentation with increasing CO. A) 10 % CO, B) 30 % CO and C) 50% CO. The CO, H_2 and CO_2 out-flow rate (mmol min^{-1}), are the average of two bioreactors, with the min and max values indicated by the colored regions.

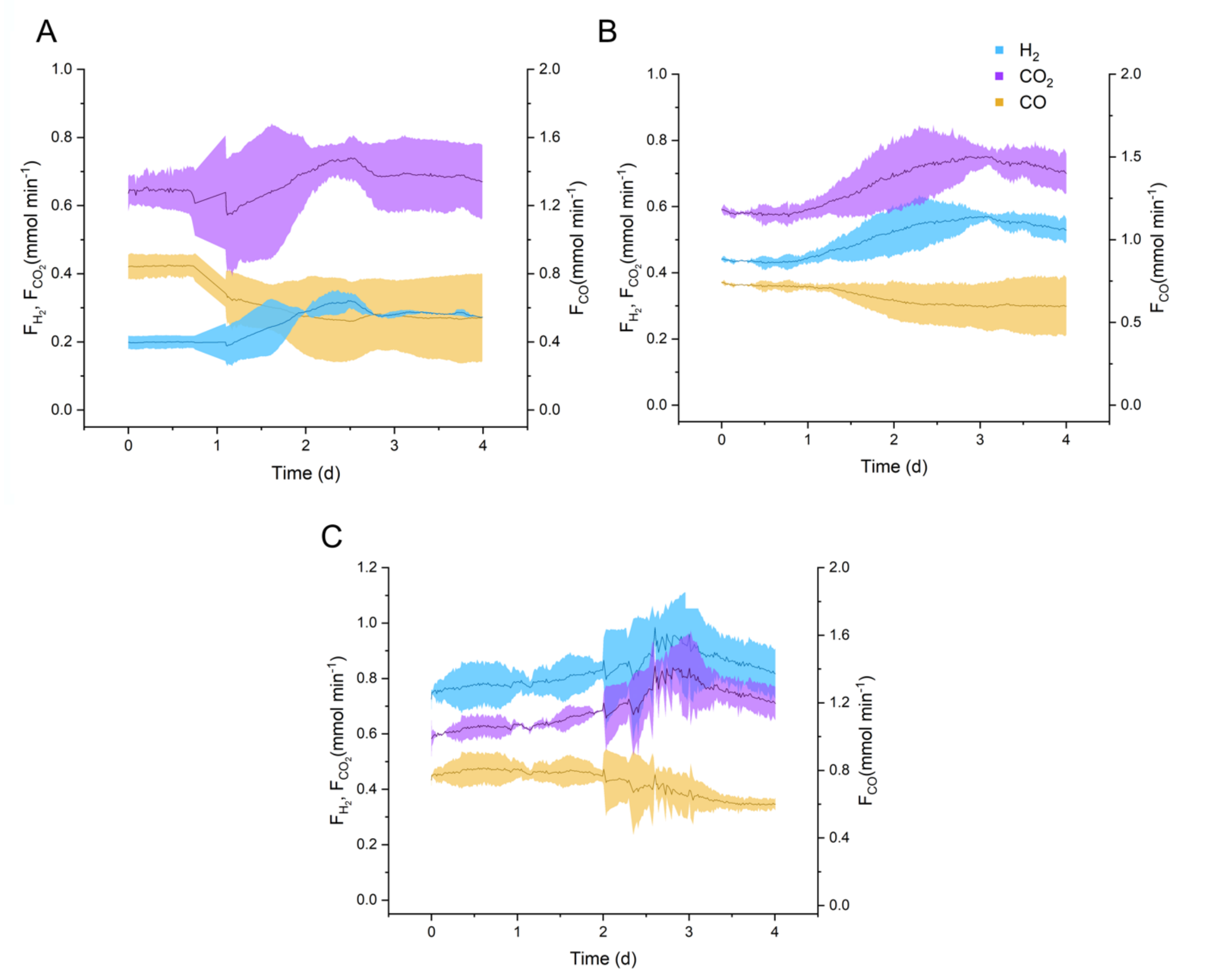

Figure S2. Syngas fermentation with increasing H_2. A) 5 % H_2, B) 12 % H_2 and C) 20 % H_2. The CO, H_2 and CO_2 out-flow rate (mmol min^{-1}), are the average of two bioreactors, with the min and max values indicated by the colored regions.

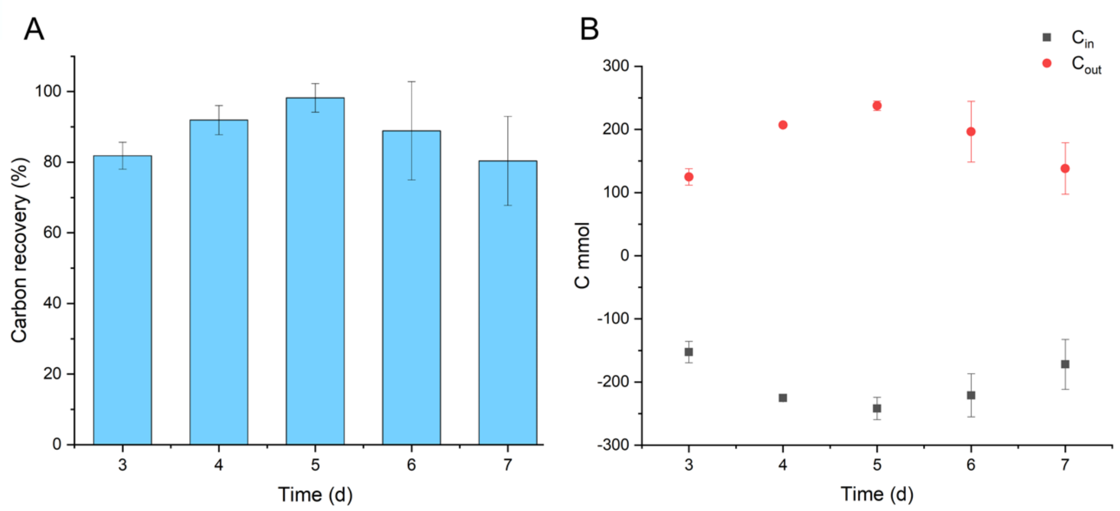

Figure S3. A) Carbon recovery for the acetate batch fermentation. B) Carbon selectivity (C mmol), C_{in} indicates the carbon in (CO, acetate), while C_{out} indicates carbon out (biomass,

formate, lactate, butyrate, propionate, valerate, iso-butyrate, iso-valerate). The data shown represent the average of two reactors, with error bars indicating the min and max values.

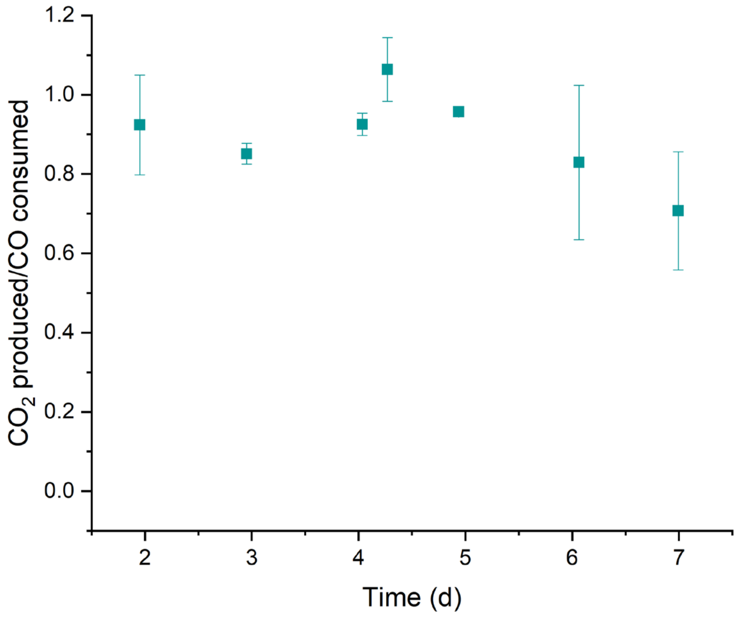

Figure S4. Quotient in terms of CO_2 produced and CO consumed during the batch fermentation with acetate as additional carbon source. The data shown represent the average of two reactors, with error bars indicating the min and max values.

Hydrogen production rate (HPR)

$$n_{H_2} = \bar{F}_{H_2} * \Delta t$$

Here, n_{H_2} are the moles of H_2, $\bar{F}_{H_2}$, is the average of the molar outflow difference (H_2 out – H_2 in) of H_2 for that interval, and Δt, the difference in sampling time.

$$V_{H_2} = n_{H_2}(mmol) * \frac{1\ mol}{1000\ mmol} * \frac{22.4\ L}{1\ mol}$$

Here, V_{H_2} is the volume of H_2 in L, taking into account that the volume of a gas, at standard temperature and pressure, is 22.4 L. The HPR was calculated using the formula:

$$HPR = \frac{V_{H_2}}{V_{media} * \Delta t}$$

Considering V_{media} as the volumeof the media in L . The intervals Δt, were designed 1 (0-4 h), 2 (4-24 h), 3 (24-48 h), 4 (48-72 h), and 5 (72-96 h).

Electron selectivity

The electron fluxes were determined using conversion factors and the quantities of each compound, as described in Equation 1.

$$e^{-}mmol_X = \dot{n}_X * eeq_X \quad (1)$$

Here, $\dot{n}_X$ represents the daily uptake rate of the substrates or the daily production rate of the products, while eeq_X is the electron equivalents for each compound.
The process selectivity towards the products was calculated with Equation 2.

$$e^{-}mol\ Selectivity\ [\%] = \frac{\sum e^{-}mmol_{Products}}{\sum e^{-}mmol_{Substrates}} * 100\% \quad (2)$$

The total daily $e^{-}mol$ was derived from the sum of electron moles contributed by carbon monoxide (CO) and glucose (Syngas fermentations) or acetate ($\sum e^{-}mmol_{Substrates}$). Similarly, the electron flow associated with the products ($\sum e^{-}mmol_{Products}$) was calculated from the sum of the daily $e^{-}mol$ from acetate, formate, lactate, and propionate. However, this calculation was only applied when these metabolites were not consumed; if they were consumed, they were instead considered substrates in the balance.

Carbon selectivity

The carbon fluxes were determined using conversion factors and the quantities of each compound, as described in Equation 3.

$$C\ mmol_X = \dot{n}_X * eeq_X \quad (3)$$

The carbon selectivity towards the products was calculated with Equation 4.

$$C\ mol\ Selectivity\ [\%] = \frac{\sum C\ mmol_{Products}}{\sum C\ mmol_{Substrates}} * 100\% \quad (4)$$

The total daily $C\ mol$ was derived from the sum of electron moles contributed by carbon monoxide (CO) and acetate ($\sum C\ mmol_{Substrates}$). Similarly, the carbon associated with the products ($\sum C\ mmol_{Products}$) was calculated with the sum of the daily $C\ mol$ from biomass, formate, lactate, butyrate, propionate, valerate, iso-butyrate, and iso-valerate.

Conversion factors

The electron balance was performed using the conversion factors in Table S1. This is based on the oxidation state of each element of the compounds. For example, for acetate (CH_3COO^-), each element has an oxidation number, for carbon is +4, hydrogen is +1, and oxygen contributes to -2 electrons. Due to the molecular formula of acetate $C_2H_4O_2$, each element´s oxidation number is multiplied by the number of atoms, for carbon, this would be 2 x 4, for hydrogen is 4 x 1 and for oxygen 2 x (-2). Everything sums (8+4+(-4)) to 8 electrons. Now, for the carbon mol calculations, this is the number of carbon atoms in each compound that is used as the conversion factor.

Table S1. Conversion factors

Compound	Molecular Weight (g/mol)	mol e^-/mol	mol C/mol
CO	28.0	2	1
CO_2	44.0	0	1
Biomass	24.6	-	1
Hydrogen	2.0	2	0
Glucose	180.1	24	6

Formate	46.1	2	1
Acetate	60.0	8	2
Lactate	90.0	12	3
Propionate	74.0	14	3

Henry's Solubility Constants in Water

The temperature dependence of Henry's law constants can be described with the van't Hoff equation:

$$\frac{d\ln H_s}{d(1/T)} = \frac{-\Delta_{sol}H}{R}$$

where Δ_{sol}H is the enthalpy of dissolution, T is the temperature, and R is the gas constant.

Table S2. Henry's law solubility constants H_s^{cp} for different gases at 25 and 55°C according to Sander (2023).

**GAS**	$-\Delta_{sol}\boldsymbol{H}/R$ (K)	$\boldsymbol{H_s^{cp}}$ at 25 °C (mol·m⁻³·Pa⁻¹)	$\boldsymbol{H_s^{cp}}$ at 55 °C (mol·m⁻³·Pa⁻¹)
CO	1300	9.7×10^{-6}	6.5×10^{-6}
**CO_2**	2400	3.3×10^{-4}	1.6×10^{-4}
**O_2**	1700	1.3×10^{-5}	7.7×10^{-6}
**H_2**	500	7.8×10^{-6}	6.7×10^{-6}
